# The discriminatory capability of existing scores to predict advanced colorectal neoplasia: a prospective colonoscopy study of 5,899 screening participants

**DOI:** 10.1038/srep20080

**Published:** 2016-02-03

**Authors:** Martin C. S. Wong, Jessica Y. L. Ching, Simpson Ng, Thomas Y. T. Lam, Arthur K. C. Luk, Sunny H. Wong, Siew C. Ng, Simon S. M. Ng, Justin C. Y. Wu, Francis K. L. Chan, Joseph J. Y. Sung

**Affiliations:** 1 Institute of Digestive Disease, Chinese University of Hong Kong, 7/F, Lui Che Woo Clinical Science Building, Prince of Wales Hospital, Shatin, NT, HKSAR; 2 School of Public Health and Primary Care, Chinese University of Hong Kong, Shatin, 4/F, School of Public Health Building, Prince of Wales Hospital, Shatin, NT, Hong Kong, HKSAR, China

## Abstract

We evaluated the performance of seven existing risk scoring systems in predicting advanced colorectal neoplasia in an asymptomatic Chinese cohort. We prospectively recruited 5,899 Chinese subjects aged 50–70 years in a colonoscopy screening programme(2008–2014). Scoring systems under evaluation included two scoring tools from the US; one each from Spain, Germany, and Poland; the Korean Colorectal Screening(KCS) scores; and the modified Asia Pacific Colorectal Screening(APCS) scores. The c-statistics, sensitivity, specificity, positive predictive values(PPVs), and negative predictive values(NPVs) of these systems were evaluated. The resources required were estimated based on the Number Needed to Screen(NNS) and the Number Needed to Refer for colonoscopy(NNR). Advanced neoplasia was detected in 364 (6.2%) subjects. The German system referred the least proportion of subjects (11.2%) for colonoscopy, whilst the KCS scoring system referred the highest (27.4%). The c-statistics of all systems ranged from 0.56–0.65, with sensitivities ranging from 0.04–0.44 and specificities from 0.74–0.99. The modified APCS scoring system had the highest c-statistics (0.65, 95% C.I. 0.58–0.72). The NNS (12–19) and NNR (5-10) were similar among the scoring systems. The existing scoring systems have variable capability to predict advanced neoplasia among asymptomatic Chinese subjects, and further external validation should be performed.

Colorectal cancer (CRC) is the third most common cancer in the world^1^, accounting for 10% of all malignancies and 8% of all cancer mortality in 2012. Its incidence is rapidly rising in both Western and Asia Pacific countries^1^,^2^. CRC screening using colonoscopy has been shown to be effective to reduce CRC mortality by 68%^3^,^4^. Both stool-based occult blood tests and colonoscopy have been recommended as primary screening modalities for CRC screening^5^,^6^, and the updated Asia Pacific Consensus Recommendations suggested that colonoscopy is the preferred choice in increased risk individuals^7^.

However, limited colonoscopy resource has been a widely recognized barrier hindering CRC screening^8^,^9^. Risk prediction models could therefore prioritize high-risk subjects to receive colonoscopy – which optimizes efficiency of resources for screening. In the past decade, a number of risk scoring systems have been designed and validated based on subjects in various countries and regions^10^,^11^,^12^,^13^,^14^,^15^,^16^,^17^. These included two scores derived from US residents^10^ and US physician, respectively;^11^ one each from Spain^12^, Germany^13^, and Poland;^14^ the Asia Pacific Colorectal Screening (APCS) score;^15^ the Korean Colorectal Screening (KCS) score;^16^ and the modified APCS score^17^. These studies prospectively recruited asymptomatic CRC screening participants, with significant risk factors identified from a derivation cohort and performance evaluated in a validation cohort. These systems included a combination of well-defined risk factors for CRC as predictive parameters, including age^12^,^18^,^19^, sex^12^,^19^,^20^, family history of CRC^12^,^21^, smoking^22^,^23^, body mass index (BMI)^19^,^24^, dietary factors^23^,^25^, and long-term use of non-steroidal anti-inflammatory drugs (NSAIDs)^23^,^26^.

Nevertheless, the discriminatory capability of these tools to predict advanced neoplasia in specific population groups remained unexplored. It has been demonstrated that the incidence and distribution of colorectal neoplasia are different among different racial and ethnic groups^27^,^28^,^29^,^30^. The original studies which published these scoring systems called for external validation in other cohorts^10^,^11^,^12^,^13^,^14^,^15^,^16^,^17^. The population of Greater China was 1.39 billion in 2013, excluding residents living in various continents in the globe. The proportion of ethnic Chinese population in the world was 20%^31^, highlighting a need to evaluate the most suitable tool for risk stratification for this ethnic group.

The objective of this study is to compare the predictive performance and resources required of these seven published risk scoring tools to detect advanced neoplasia in a large Chinese population. These findings could inform the predictive capability of the existing scoring systems and the resources required to identify subjects with advanced neoplasia-i.e. whereby colonoscopy is warranted.

## Methods

A bowel cancer screening centre was established in Hong Kong in 2008, which provides free-of-charge CRC screening services for eligible Hong Kong citizens^32^,^33^. The centre is accessible to all Hong Kong residents. Following several media announcements, we recruited subjects for free screening services by registrations via telephone, e-mail, fax or walk-in. The present evaluation included all screening participants who received colonoscopy in the study period 2008-2014. The Clinical Research Ethics Committee of the Chinese University of Hong Kong approved the study (protocol CRE-2008.404), and the methods were carried out in accordance with the approved guidelines. All participants provided informed consent before enrolling into the study, and were invited to visit the centre before screening.

### Screening Participants

Upon centre visits, two independent health educators checked for subject eligibility. These eligibility criteria included: (i) age 50–70 years; (ii) the absence of any current and previous CRC symptoms, such as haematochezia, tarry stool, anorexia or change in bowel habit in the past 4 weeks, or unintentional weight loss of greater than 5 kg in the past 6 months; and (iii) not having undergone any CRC screening tests in the past 5 years. Exclusion criteria consist of personal history of CRC, colorectal adenoma, inflammatory bowel disease and the presence of medical conditions which were contraindications for colonoscopy, like cardiopulmonary insufficiency and the use of double antiplatelet therapies.

### Screening Colonoscopy

All study participants were explained about the nature, benefits and risk of colonoscopy before the procedures. We used polyethylene glycol (Klean-Prep^R^, Helsinn Birex Pharmaceuticals Ltd, Ireland) as a standard bowel preparation regime for all participants, who were reminded of colonoscopy attendance before they left the centre. A team of experienced physicians and colorectal surgeons performed all colonoscopy procedures in the endoscopy centres affiliated with the University. The sedation regimen used included midazolam 2.5 mg (Groupe Panpharma, France) and meperidine 25 mg (Martindale Pharmaceuticals, United Kingdom). Further doses of these drugs were administered according to the subject’s level of discomfort. We used air insufflation and aimed for cecal intubation, aiming for a withdrawal time of ≥6 minutes in accordance with the current quality indicators for colonoscopy^34^. All colorectal lesions were removed and biopsied as deemed appropriate by the endoscopists. We sent all the biopsied samples to an accredited laboratory for gross and microscopic examination. Advanced neoplasia was defined as CRC or any colorectal adenoma which has (1). a size of ≥10 mm in diameter; (2). high grade dysplasia; (3). villous or tubulovillous histologic characteristics, or any combination thereof^19^. In the presence of multiple lesions, the most advanced characteristic was assigned.

### Evaluation of the existing scoring systems: outcome variables and statistical analysis

From a thorough literature review, seven studies which devised and validated scoring systems based on elementary clinical information to predict advanced neoplasia were identified^10^,^11^,^12^,^13^,^14^,^15^,^16^,^17^. Table 1 summarizes the key feature, predictor variables, and the computational algorithm of each scoring system. We defined the threshold for colonoscopy referral as the cut-off score where: (1). the subjects were classified as high or very high risk; or (2). the subjects in the initial development and validation of the scoring system were found to be at a specific risk level which was just higher than that of the whole cohort in the respective study.

For the US Physician health survey^11^, only male subjects in our cohort were included, since the US survey exclusively consists of male physicians. In the German cohort evaluated by Tao and colleagues^13^, we only included non-smokers, non-drinkers, as we do not have detailed information on pack years and drinking frequency in our cohort. For the Poland system devised by Kaminski *et al*.^14^, only non-smokers aged 66 years or below from our participants were included. The original APCS scoring system developed by Yeoh and colleagues has been extensively evaluated in the Asia-Pacific countries and the c-statistics was found to be 0.64 ( ± 0.04)^15^. A modified version of the APCS scoring system has been devised and validated in 7,463 subjects from 11 Asian cities, with a c-statistics of 0.65 (95% C.I. 0.58–0.72)^17^. It incorporated body mass index as a predictor variable in addition to age, gender, smoking and family history of CRC. In this study we studied the modified APCS system.

The proportion of subjects referred for colonoscopy in our cohort was delineated when different scoring systems were applied – and each was compared with the modified APCS scoring system using McNemar test. The accuracy of all the prediction strategies to detect advanced neoplasia was evaluated, including the sensitivity, specificity, and positive predictive values (PPVs) and negative predictive values (NPVs). The discriminatory ability for prediction was computed for each scoring model, presented as the concordance (c-) statistics. The c-statistics was used to measure the discriminatory power between those with and without advanced neoplasia^35^, which is identical to the Area Under the Curve (AUC) for binary logistic regression models. The statistics considered all pairs of subjects, and computed the proportion of pairs in which the model accurately predicted a higher likelihood of advanced neoplasia for subjects categorized as high risk. Similar to the approach adopted by Imperiale *et al*.^36^, a c-statistic of 0.7–0.8 was regarded as good discrimination, whilst a value >0.8 indicated excellent discrimination. A c-statistics between 0.6–0.7 had some clinical value whereas c-statistics <0.6 had no clinical value. We employed the deLong test to compare the AUCs of the seven systems. Using the modified APCS scoring system as the comparison group, each system was evaluated according to their ability to accurately classify subjects into high vs. low risk group. This is presented as the Net Reclassification Index (NRI), defined as the sum of differences in proportions of correct reclassification minus incorrect reclassification. A positive NRI indicates more accurate classification of risk for advanced neoplasia by the assessed system than the modified APCS system; whilst a negative NRI indicates less accurate classification of the risk by the assessed system than the modified APCS system.

The resources required to detect advanced neoplasia was estimated by the Number Needed to Screen (NNS) and the Number Needed to Refer (NNR) for colonoscopy to detect one advanced neoplasia. The NNS was the total number of subjects in each subgroup divided by the number of subjects referred for colonoscopy and detected as having advanced neoplasia, according to each risk stratification system. The NNR was the number of subjects referred for colonoscopy divided by the number of subjects referred for colonoscopy and detected as having advanced neoplasia. The Statistical Package for Social Sciences version 19.0 was used for all data analysis. P values < 0.05 were considered statistically significant.

## Results

### Participant characteristics

From a total of 5,899 eligible screening participants, the average age was 57.7 years (SD 4.9) (Table 2). Male subjects consist of 47.1%, and the proportion of smokers and alcohol drinkers was 8.3% and 9.7%, respectively. Of all participants, 1,700 (29.2%) had BMI ≥ 25 kg/m^2^, 847 (14.4%) had a family history of CRC in a first-degree relative, and the proportion of subjects self-reported as having diabetes, hypertension, and current NSAID use was 7.6%, 23.2% and 4.7%, respectively. There were 25 CRC (0.4%) and 339 (5.7%) advanced adenomas. The characteristics of the screening participants are summarized in Table 2.

### Proportions of colonoscopy referral

The proportion of subjects referred for colonoscopy was the highest with the KCS scores (27.4%, 95% C.I. 26.3%–28.6%), followed by the Spain scores (25.5%, 95% C.I. 24.4%–26.7%), and the modified APCS scores (21.4%, 95% C.I. 20.3%–22.5%). The proportion of colonoscopy referral by applying each scoring system (p < 0.001) was significantly different from that of the modified APCS scores (Table 3).

### Performance of the scoring systems

The c-statistics of the scoring systems ranged from 0.56 (95% C.I 0.48–0.64) [the Spain system] to 0.65 (95% C.I. 0.58–0.72) [the modified APCS system] (Table 4). The sensitivity of these systems ranged from 0.04–0.44 and the specificity was moderate to high (range 0.74–0.99). All of them had low PPVs (range 0.10–0.19) and high NPVs (range 0.92–0.96). The deLong tests showed that the AUCs of all the six scores, other than the modified APCS score, had no statistically significant difference.

Using the modified APCS scoring system as a comparator, the NRI of the Spain (−3.3%, 95% C.I. −8.0% to 1.4%), Germany (−0.5%, 95% C.I. −6.4% to 5.5%), Poland (1.2%, 95% C.I. −2.7% to 5.2%) and the KCS (−2.7%, 95% C.I. −7.2% to 1.9%) was statistically similar (all p > 0.05) (Table 5). The US-Seattle (−8.1%, 95% C.I. −13.1% to −3.1%, p = 0.001) and the US physician health survey (−20.4%, 95% C.I. −27.2% to −13.6%, p < 0.001) classified advanced neoplasia less accurately than the modified APCS scores (Table 5).

### Colonoscopy resources

The NNS ranged from 12 (95% C.I. 6–21) of the US physician health survey to 19 (95% C.I. 11–30) of the Germany and Poland scores (Table 6). The NNR ranged from 5 (95% C.I. 2–12) of the US physician health survey to 10 (95% C.I. 5–18) of the Spain, Poland, and KCS systems. There were no significant differences of both NNS and NNR among the various scoring systems (Table 6).

## Discussion

This study compared the performance of seven existing prediction models in a Chinese population. From 5,899 asymptomatic subjects, 6.1% had advanced neoplasia. The proportion of screening participants referred for colonoscopy was the highest using the KCS and the Spain scores, and the lowest using the Germany and the US Physician Health Survey criteria. The scoring systems had variable discriminatory ability to predict the risk of advanced neoplasia (c-statistics ranged from 0.56–0.65). The modified APCS score seemed a preferable system to classify high risk subjects based on its highest c-statistics. These findings implied that prediction tools for advanced neoplasia may need further external validation to evaluate their generalizability.

The modified APCS scoring system demonstrated a higher discriminatory ability to detect advanced neoplasia and improvements in risk prediction compared with two other tools. There is a relatively high proportion of Chinese subjects represented in this score (5,795 out of 7,463; 77.6%), whilst the other subjects were recruited in Korea (4.0%), Malaysia (5.8%), the Philippines (0.7%), Singapore (0.9%), Thailand (4.2%), Japan (5.3%), Brunei (1.1%) and Pakistan (0.4%). Also, BMI was included as a predictive variable – a parameter missing in the original APCS score^15^. In addition, despite the lowest c-statistics of the KCS scoring system^16^, its NNS and NNR were similar to other scores. It might be that the difference in c-statistics with other systems was so small to observe a clinically significant difference in NNS/NNR. Future studies are needed to explore further rooms for improving the discriminatory capability of the KCS scoring system.

This is the first large-scale study which evaluated the predictive ability and colonoscopy resources required when the existing prediction tools were applied in a Chinese population. The evaluation is unique as we did not only evaluate these scoring systems by their concordance statistics. The approach of solely relying on discrimination measures has been criticized, since calibration (i.e. the agreement between predicted and observed risk) is also a crucial aspect of model performance^37^,^38^. Most importantly, comparing c-statistics lacks an apparent clinical interpretation, such as how patient classification would improve with inclusion or exclusion of other predictors in risk scoring systems. Reclassification has recently become a popular approach for comparing improvement among risk scoring systems for diagnosing common diseases^39^,^40^,^41^. A model is considered better when individuals who have subsequently developed the disease and those who have not developed the disease are reclassified to a higher risk category and to a lower risk category, respectively.

This study highlighted a need to modify existing tools with c-statistics <0.60 to risk stratify subjects for colonoscopy screening – and is clinically important as identification of advanced neoplasia enables secondary prevention by polypectomy^19^,^42^. The estimation of individual risk for advanced neoplasia may facilitate informed, shared decision making process about screening as part of patient-centred care^43^. Nevertheless, there are some limitations which should be addressed. Firstly, we invited screening participants who were self-referred following media announcements. They may be different from the general public – therefore our subjects are more representative of patients who volunteered as screening participants. The self-selected population included in the present cohort had low prevalence of smoking or alcohol drinking, and high proportion of them had family members suffering from CRC – which may reflect the better health-consciousness when compared with the general population. One may also anticipate that a revised methodology to recruit subjects using a population-based, random sampling approach may meet with a high refusal rate. Secondly, the German and Poland systems^13^,^14^ excluded some of our subjects and this might have introduced biases, since in our original cohort comprehensive data on number of pack years among smokers and frequency of alcohol drinking were not collected. The original Kaminski score^14^ was developed from screening subjects aged between 40–66 years, thus limiting the number of subjects which could be included for external validation. In addition, we are unable to validate some scoring systems, including the prediction model constructed by Cai and colleagues in China, which has been demonstrated as having better discrimination in previous evaluations^44^. Among eight parameters, four variables require dietary recall of green vegetable, prickled food, fried food, and white meat intake. Detailed information of these food items were not collected in our cohort. Another system developed by Law and colleagues in Malaysia^45^ was mainly reserved for symptomatic patients, and none of our subjects in our study was categorized as high risk, as they were all asymptomatic. Other scoring systems required detailed information on family history, physical activity, leisure time vigorous activity, dietary intake, the use of hormone replacement therapy, and folate consumption^46^,^47^,^48^,^49^,^50^. Critics might argue that exclusion of high-risk subjects (smokers and drinkers) and low-risk subjects (women) might lead to altered estimates of the discriminatory ability of these three scoring systems. Furthermore, the choice of cut-off value for each prediction system was based on the recommendation from the respective original article. It still remains to be explored whether addition of more variables could further improve the scoring systems, such as lifestyle measures (like dietary intake; smoking and alcohol drinking) and medical conditions known to be risk factors for advanced neoplasia (like diabetes and central obesity measured by waist circumference). Besides, some variables like age and BMI could be analyzed by treating them as continuous variables which might enhance the concordance statistics. Finally, the nature of these risk models may apply to lifetime or longer-term prediction of risk for ACN, and the cross-sectional nature of the present study might benefit by further prospective colonoscopy follow-up for additional risk score validation.

In summary, these findings suggested that in the absence of newer prediction tools, the modified APCS system could be useful to risk-stratify ethnic Chinese subjects. The formulation and implementation of a higher-performing scoring system may optimize the efficiency of screening resources and prioritize high-risk subjects for colonoscopy. Future studies may evaluate the performance of these scoring systems where the same cut-off values for absolute risk of ACN were applied, and target on devising scores using additional variables.

## Additional Information

**How to cite this article**: Wong, M. C. S. *et al*. The discriminatory capability of existing scores to predict advanced colorectal neoplasia: a prospective colonoscopy study of 5,899 screening participants. *Sci. Rep*. **6**, 20080; doi: 10.1038/srep20080 (2016).

## Figures and Tables

**Table 1:** Existing scoring systems for risk prediction of advanced neoplasia.

	Investigators	Scoring algorithm	Ref.
Scoring systems evaluated in this study			
US Seattle	Lin *et al*. (2006)	- Age (<55: 0; 55–59: 1; 60–64: 2; >64: 3)	10
		- Sex (Male: 1; female: 0)	
		- Family history (No: 0; only second degree relative with CRC: 1; first degree relative with CRC: 2)	
US physician health survey	Driver *et al*. (2007)	- Age (50–59: 2; 60–69: 4; ≥70: 6)	11
		- Smoking status (Yes = 1; No = 0),	
		- Alcohol drinking (Yes = 1) and	
		- Body mass index (<25 = 0; 25–29.9: 1; ≥30: 2)	
Spain	Betes *et al*. (2003)	- Age (≤50: 0; 51–60: 1; 61–70: 2; 71–80; 3; >80: 4)	12
		- Sex (Male: 2; Female: 0)	
		- BMI (≤25: 0; 25–35: 1; >35: 2)	
Germany	Tao *et al*. (2014)	- Sum of the scores from the parameters below:	13
		- Age (multiplied by 6);	
		- Sex (male: 104; Female: 0);	
		- No. of first-degree relatives with a history of CRC (multiplied by 35)	
		- Cigarette smoking (number of pack years);	
		- Alcohol consumption (gram/day);	
		- Red meat consumption (>1 time/day: multiplied by 47)	
		- Ever regular use [at least 2 times/wk for at least 1 y] of nonsteroidal anti-inflammatory drugs (Yes: minus 31)	
		- Previous colonoscopy (Yes: minus 147)	
		- Polyp history (Yes: 187)	
Poland	Kaminski *et al*. (2014)	- Age (40–49: 0; 50–54: 1; 55–59: 2; 60–66: 3)	14
		- Sex (Male: 2; Female: 0)	
		- Family history of CRC (One first-degree relative, age ≥60 years: 1; One first-degree relative, age <60 years: 2; Two first-degree relatives: 2)	
		- Smoking history (None or <10 pack-years: 0; ≥10 pack-years: 1)	
		- BMI (<30 kg/m^2^: 0; ≥30 kg/m^2^: 1)	
Korean Colorectal Screening (KCS)	Kim *et al*. (2014)	Age (<50: 0; 50–69: 2; ≥70: 4),	16
		Sex (male: 1; female: 0)	
		Body mass index (<25: 0; ≥25: 1)	
		Smoking (non-smoker: 0; current or past smoker: 1),	
		Family history of CRC (yes: 1; no: 0)	
Modified APCS	Sung *et al*. (2014)	Age (40–49: 0; 50–59: 1; ≥60: 2)	17
		Sex (Male: 1; Female: 0)	
		Family history present for a first-degree relative (Present: 1)	
		Smoking history (Current or past: 1)	
		BMI (<23 kg/m^2^: 0; ≥23 kg/m^2^: 1)	
Scoring systems not evaluated in this study due to lack of relevant information			
US Boston	Kastrinos *et al*. (2009)	(i) "Do you have a first-degree relative with CRC or LS-related cancer diagnosed before age 50?"	46
		(ii) "Have you had CRC or polyps diagnosed before age 50?"	
		(iii) "Do you have ≥3 relatives with CRC?"	
The National Institutes of Health –American Association of Retired Persons (AARP) diet and health study	Park *et al*. (2009)	Age	47
		Gender	
		Screening results in the past 3 years	
		Number of relatives with CRC	
		Physical Activity (>4 h/week; >2 and ≤4 h/week; >0 and ≤2 h/week; 0 h/week)	
		Aspirin/NSAID use	
		Vegetable intake (servings <5/day; ≥5/day)	
		BMI (<25; ≥25 and <30; ≥30 kg/m^2^)	
		Cigarette smoking (0; 1–10; 11–19; ≥20/day)	
		Oestrogen Status (negative vs. positive)	
Universities of Utah, Minnesota, and the Kaiser Permanente Medical Care Program (KPMCP) of Northern California (Oakland, CA).	Freeman *et al*. (2009)	Polyp history in the last 10 years,	48
		History of CRC in first-degree relatives,	
		Aspirin and NSAID use,	
		Cigarette smoking,	
		Body mass index (BMI),	
		Current leisure-time vigorous activity	
		Vegetable consumption.	
		Previous sigmoidoscopy/colonoscopy,	
		Hormone-Replacement Therapy (HRT)	
		Oestrogen exposure on the basis of menopausal status	
Nurses’ Health Study	Wei *et al*. (2009)	Family history of CRC	49
		Cigarette smoking before age 30 years	
		Tallness and Body Weight	
		Current postmenopausal hormone use	
		Physical activity level	
		Taking aspirin	
		Having screened for CRC	
		Consumption of red or processed meat	
		Consumption of folate	
Japan Public Health Center-based (JPHC) Prospective Study Cohort II	Ma *et al*. (2010)	Age,	50
		BMI,	
		Alcohol consumption,	
		Smoking status	
		Daily physical activity level	

**Table 2:** Characteristics of individuals included in the analysis (N = 5,899).

Characteristic	**N (%)**
Age, years, mean (SD)	57.73 (4.93)
Age	
<50	11 (0.2)
50–55	2240 (38.0)
56–60	1940 (32.9)
61–65	1214 (20.6)
66–70	482 (8.2)
>70	12 (0.2)
Sex	
Male	2777 (47.1)
Female	3122 (52.9)
Smokers (Current or past)	488 (8.3)
Alcohol Drinkers	573 (9.7)
BMI (kg/m^2^)*	
<23	2640 (45.3)
23–24.9	1490 (25.6)
≥25	1700 (29.2)
Family history of CRC (first degree relatives)	847 (14.4)
Diabetes Mellitus	446 (7.6)
Hypertension	1367 (23.2)
Use of Non-steroidal anti-inflammatory drugs (NSAIDs)	279 (4.7)
Most advanced finding:	
Colorectal cancer	25 (0.4)
Proximal	8 (0.1)
Distal	16 (0.3)
Both	1 (0.02)
Advanced adenoma	339 (5.7)
Proximal	126 (2.1)
Distal	194 (3.3)
Both	19 (0.3)
Nonadvanced adenoma	1554 (26.3)
Proximal	624 (10.6)
Distal	682 (11.6)
Both	248 (4.2)

*69 missing.

**Table 3:** Individuals referred for colonoscopy according to each scoring system.

	**High risk criteria**	**N**	**Referred for colonoscopy (%)**	**95% CI**	**P^§^**
US (Seattle)^1^	≥4 (Max. = 6)	5899	829 (14.1)	13.2–15.0	<.001
US physician health survey^2^	≥7 (Max. = 10)	2761	43 (1.6)	1.2–2.1	<.001
Spain^3^	≥4 (Max. = 8)	5830	1487 (25.5)	24.4–26.7	<.001
Germany^4^	≥470.78	3281	366 (11.2)	10.1–12.3	<.001
Poland^5^	≥5 (Max. = 9)	5107	890 (17.4)	16.4–18.5	<.001
Korean Colorectal Screening (KCS)^6^	≥4 (Max. = 8)	5830	1598 (27.4)	26.3–28.6	<.001
Modified APCS^7^	≥4 (Max. = 6)	5830	1159 (19.9)	18.9–20.9	N/A

^1^Lin *et al*. (2006) Gastroenterology 131:1011-1019.

^2^Driver *et al*. (2007). Am J Med 120:257-263(*Only male subjects were included).

^3^Betes *et al*. (2003). Am J Gastroenterol 98:2648-2654 (*69 subjects with BMI missing).

^4^Tao *et al*. (2014). Clin Gastrointest Hepatol 12:478-485(*Only non-smokers, non-drinkers and those not taking lots of meat were included)

^5^Kaminski *et al*. (2014). Gut 63:1112-1119(*Only non-smokers aged 66 or below were included).

^6^Kim *et al*. (2014). J Clin Gastroenterol Feb 27. [Epub ahead of print] (*69 subjects with BMI missing).

^7^Sung JJ, Wong MC, Tsoi KK (2014). Gastroenterology 2014; 146:S-730 (*69 subjects with BMI missing).

^§^Comparison between modified APCS score and each scoring system was performed by pair-wise χ^2^ test (two-sided).

**Table 4:** Performance characteristics of the various scoring systems.

	**c-statistics (95% C.I.)**	**Sensitivity (95% CI)**	**Specificity (95% CI)**	**PPV (95% CI)**	**NPV (95% CI)**
US (Seattle)^1^	0.601 (0.530–0.673)	0.25 (0.21–0.30)	0.87 (0.86–0.88)	0.11 (0.09–0.13)	0.95 (0.94–0.95)
US physician health survey^2^	0.589 (0.507–0.670)	0.04 (0.02–0.07)	0.99 (0.98–0.99)	0.19 (0.09–0.34)	0.92 (0.91–0.93)
Spain^3^	0.563 (0.483–0.642)	0.41 (0.36–0.46)	0.76 (0.74–0.77)	0.10 (0.08–0.12)	0.95 (0.94–0.96)
Germany^4^	0.620 (0.548–0.691)	0.24 (0.18–0.31)	0.90 (0.88–0.91)	0.11 (0.08–0.15)	0.95 (0.95–0.96)
Poland^5^	0.607 (0.533–0.681)	0.32 (0.27–0.38)	0.83 (0.82–0.84)	0.10 (0.08–0.12)	0.96 (0.95–0.96)
Korean Colorectal Screening (KCS)^6^	0.569 (0.491–0.647)	0.44 (0.39–0.49)	0.74 (0.72–0.75)	0.10 (0.08–0.11)	0.95 (0.95–0.96)
Modified APCS^7^	0.650 (0.576–0.724)	0.39 (0.34–0.44)	0.81 (0.80–0.82)	0.12 (0.10–0.14)	0.95 (0.95–0.96)

^1^Lin *et al*. (2006) Gastroenterology 131:1011-1019.

^2^Driver *et al*. (2007). Am J Med 120:257-263 (*Only male subjects were included).

^3^Betes *et al*. (2003). Am J Gastroenterol 98:2648-2654.

^4^Tao *et al*. (2014). Clin Gastrointest Hepatol 12:478-485(*Only non-smokers, non-drinkers and those not taking lots of meat were included).

^5^Kaminski *et al*. (2014). Gut 63:1112-1119 (*Only non-smokers aged 66 or below were included).

^6^Kim *et al*. (2014). J Clin Gastroenterol Feb 27. [Epub ahead of print].

^7^Sung *et al*. (2014). Gastroenterology 2014; 146:S-730.

CI = confidence interval; NPV = negative predictive value; PPV = positive predictive value.

**Table 5:** The Reclassification performances of each risk scoring system

**Risk in Modified APCS**	**Low Risk**	**High Risk**	**Reclassified (%)**	**NRI (95% C.I.)**	**p values**
US-Seattle^1^					
Low risk	4,410	261	5.6	−8.1%	0.001
High risk	600	559	51.8	(−13.1% to −3.1%)	
US physician health survey^2^					
Low risk	1,685	2	0.1	−20.4%	<0.001
High risk	1,033	41	96.2	(−27.2% to −13.6%)	
Spain^3^					
Low risk	4,042	629	13.5	−3.3%	0.165
High risk	301	858	26.0	−8.0% to 1.4%	
Germany^4^					
Low risk	2,713	158	5.5	−0.5%	0.873
High risk	135	229	37.1	(−6.4% to 5.5%)	
Poland^5^					
Low risk	4,071	298	6.8	1.2%	0.541
High risk	91	585	13.5	(−2.7% to 5.2%)	
Korean Colorectal Screening (KCS)^6^					
Low risk	3980	691	14.8	−2.7%	0.254
High risk	252	907	21.7	(−7.2% to 1.9%)	

^1^Lin *et al*. (2006) *Gastroenterology* 131:1011-1019

^2^Driver *et al*. (2007). *Am J Med* 120:257-263 (*Only male subjects were included)

^3^Betes *et al*. (2003). *Am J Gastroenterol* 98:2648-2654

^4^Tao *et al*. (2014). *Clin Gastrointest Hepatol* 12:478-485(*Only non-smokers, non-drinkers and those not taking lots of meat were included)

^5^Kaminski *et al*. (2014). *Gut* 63:1112-1119 (*Only non-smokers aged 66 or below were included)

^6^Kim *et al*. (2014). *J Clin Gastroenterol* Feb 27. [Epub ahead of print]

NRI: Net Reclassification Index. It is the sum of differences in proportions of correct reclassification minus incorrect reclassification. All scoring systems were compared with the modified APCS scoring system published by Sung *et al*. (2014). Gastroenterology 2014; 146:S-730. A positive NRI indicates more accurate classification of risk for advanced neoplasia by the modified APCS than the assessed system; whilst a negative NRI indicates less accurate classification of the risk by the assessed system than the modified APCS system.

**Table 6:** Colonoscopy resources required for each risk scoring system.

**Scoring system**	**NNS N (95% C.I.)**	**NNRN (95% C.I.)**
US (Seattle)^1^	16 (9–26)	9 (4–17)
US physician health survey^2^	12 (6–21)	5 (2–12)
Spain^3^	16 (9–26)	10 (5–18)
Germany^4^	19 (11–30)	9 (4–17)
Poland^5^	19 (11–30)	10 (5–18)
Korean Colorectal Screening (KCS)^6^	16 (9–26)	10 (5–18)
Modified APCS^7^	16 (9–26)	8 (3–16)

NNS: Number needed to screen with colonoscopy to detect one advanced neoplasm; NNR: Number needed to refer for colonoscopy to detect one advanced neoplasm; CI = confidence interval

^1^Lin *et al*. (2006) Gastroenterology 131:1011-1019.

^2^Driver *et al*. (2007). Am J Med 120:257-263 (*Only male subjects were included).

^3^Betes *et al*. (2003). Am J Gastroenterol 98:2648-2654.

^4^Tao *et al*. (2014). Clin Gastrointest Hepatol 12:478-485(*Only non-smokers, non-drinkers and those not taking lots of meat were included).

^5^Kaminski *et al*. (2014). Gut 63:1112-1119 (*Only non-smokers aged 66 or below were included).

^6^Kim *et al*. (2014). J Clin Gastroenterol Feb 27. [Epub ahead of print].

^7^Sung *et al*. (2014). Gastroenterology 2014; 146:S-730.
